# Glutathione: A Key Regulator of Extracellular Matrix and Cell Death in Intervertebral Disc Degeneration

**DOI:** 10.1155/2024/4482642

**Published:** 2024-10-01

**Authors:** Fudong Li, Shaofei Li, Yangyang Shi, Feng Lin, Lining Rui, Jiangang Shi, Kaiqiang Sun

**Affiliations:** ^1^ Department of Orthopedic Surgery Shanghai Changzheng Hospital Naval Medical University, Road No. 415, Fengyang, Shanghai 200003, China; ^2^ Department of Orthopedic Surgery The Affiliated Yantai Yuhuangding Hospital of Qingdao University, Yuhuangding East Road No. 20, Yantai 264000, China; ^3^ Department of Spinal Surgery Wujin Hospital of Traditional Chinese Medicine, Changzhou 213161, Jiangsu, China; ^4^ Department of Orthopaedic Surgery Naval Medical Center Naval Medical University, Road No. 800, Xiangyin, Shanghai 200433, China

## Abstract

Intervertebral disc degeneration (IVDD) is a degenerative disease accompanied by the loss of nucleus pulposus cells and the degradation of extracellular matrix (ECM), which tends to be associated with lower back pain. The ECM and various types of cell death in IVDD are regulated by multiple factors, such as inflammatory responses and oxidative stress. The glutathione (GSH) redox system is the most important antioxidant defense system in cells. GSH is one of the most abundant thiol antioxidants in mammalian cells, which functions directly and indirectly by scavenging peroxides through the GSH redox system. In these reactions, GSH is oxidized by electrophilic substances, such as reactive oxygen species and free radicals, to form glutathione disulfide to exert antioxidative effects. It has been reported that GSH can protect cells against the damage of oxidative stress and various pathophysiological stimulus that can lead to different types of cell death. In addition, it was reported that the level of GSH widely participates in apoptosis, autophagy, ferroptosis, and oxidative stress in many diseases including osteoarthritis and IVDD. Therefore, we summarized the effects of GSH on ECM metabolism and cells' functions during IVDD. In addition, we summarized the regulatory effects of small molecule compounds on GSH to explore potential ways to regulate the level of GSH. Better understanding the underlying role of GSH in regulating IVDD will facilitate the goal of preventing and retarding the progress of IVDD in the future.

## 1. Introduction

Low back pain (LBP) is a global health concern with a high morbidity, which is the leading cause for job disability worldwide [1]. It was reported that almost 80% of the individuals may experience LBP in their whole lifetime [2]. Although, there is often confusion regarding the etiology of LBP, it has been believed that intervertebral disc degeneration (IVDD) is the major contributor. Normally, the intervertebral disc (IVD) is aneural and avascular. Nevertheless, nerve fibers would ingrowth in the inner layers of the annulus fibrosus (AF) and even the nucleus pulposus (NP) during IVDD [3, 4].

IVDD is a complex biological process and its mechanisms remain elusive. It has been believed that abnormal loss of nucleus pulposus cells (NPCs) and excessive degradation of extracellular matrix (ECM) are the main features of IVDD [5]. Cell death can occur in several ways, including apoptosis, autophagy, and ferroptosis, which has been proved to be associated with IVDD [6, 7, 8]. Among the numerous risk factors, oxidative stress plays an essential role in aggravating or even triggering IVDD. It has been proved that IVDD could be attenuated by inhibiting oxidative stress in mouse, which has been proved to be relevant to the regulation of ferroptosis, apoptosis, and autophagy [9, 10, 11, 12]. Additionally, it was reported that the degradation of ECM can be ameliorated by inhibiting oxidative stress in ECM-degraded diseases such as osteoarthritis (OA) and IVDD [13, 14]. Therefore, we believe that oxidative stress plays an essential role in the development of IVDD.

As one of the most important antioxidants, glutathione (GSH) has been proved to not only exert powerful protective effect on cells affected by oxidative stress but also to protect cells against various pathological damages, such as apoptosis, autophagy, and ferroptosis, in many diseases. Jang et al. [15] ever reported that inhibiting two carrier proteins including the dicarboxylate carrier and the oxoglutarate carrier could aggravate ferroptosis through increasing the level of mitochondrial reactive oxygen species (ROS) and decreasing the level of GSH. In addition, Cho et al. [16] reported that cystamine caused apoptosis through GSH depletion. Sun et al. [17] reported that GSH depletion can lead to ferroptosis, autophagy, and cell senescence in retinal pigment epithelial cells. Additionally, Yang et al. [18] reported that the therapeutic effects of hyaluronic acid were significantly enhanced by the supplement of GSH in treating OA, and the study proved that GSH improved the antioxidant capacity and lowered the expression of pro-inflammatory cytokines in human fibroblast-like synoviocytes. Cheng et al. [19] reported that 100 *μ*M of GSH can rescue CDGSH iron sulfur domain 2 deficiency (Cisd2^−/−^) chondrocytes against oxidative stress through inhibiting inflammation and apoptosis in OA. Collectively, GSH plays a central role in protecting various cells from oxidative stress in many diseases. Because there are accumulating reports regarding oxidative stress in the development of IVDD, we believe that GSH possesses potent potential in protecting IVD against oxidative stress-induced cell death and ECM degradation.

In this review, we summarized the role of GSH in the regulation of IVDD. First, we reviewed previous studies regarding the effects of GSH on the balance between synthesis and degradation in the IVD. Then, we discussed the underlying mechanisms about GSH-mediated protective effects on cell proliferation, apoptosis, autophagy, and ferroptosis induced by oxidative stress. Furthermore, we reviewed the small molecule compounds that participated in regulating the level of GSH in various diseases. Taken together, studying GSH is significant in the context of IVDD because it plays a crucial role in protecting IVD cells from oxidative stress-induced cell death and ECM degradation. Understanding the protective mechanisms of GSH can lead to potential therapeutic strategies for mitigating the progression of IVDD.

## 2. IVD and IVDD

The IVD is a fibrocartilaginous structure consisting of NP tissue encircled by the AF tissue, sandwiched between cartilaginous and bony endplates [20], which plays an essential role in transmitting compressible load and trunk flexibility [21]. NP tissue derived from the notochord is mainly a hypoxic and avascular. NP is highly cellular, but the cell density in the NP tissues is relatively low in the stage of the adult [22]. NPCs arrange mainly in clusters and tend to be separated by ECM with abundant aggrecan and type Ⅱ collagen (COLII). The NPCs possess functional mitochondria and Golgi system that produce ECM proteins [23]. Aggrecan renders the IVD a high osmotic pressure. In addition, because the oxygen tension within IVD is relatively low, NPCs mainly rely on the glycolytic pathway to gain ATP [23]. The AF tissue can be divided into the inner fibrocartilaginous region and the outer fibrous zone [24]. The outer AF is mainly composed of type Ⅰ collagen fibers and the inner AF contains type Ⅰ and Ⅱ collagen fibers. Cells in the outer AF tissue are elongated and fusiform, while the cells in the inner AF tissue are spherical in shape and this is similar to NPCs [25]. Endplate, the thin layer of cartilage, frequently thins with age. The chondrocytes in endplate are embedded in ECM with plenty of aggrecan and COLII. Notably, vascular channels penetrate the cartilage in child, whereas the vessels gradually become obliterated with age [26], and the nutrient supply to the cartilage and the disc can be affected.

The degeneration of the IVD is associated with aging. It was reported that the incidence of IVD degeneration was 1%–6% among children aged 7–10 years, 18% among adolescences, and 28%–42% in individuals between 40 and 69 years old [27, 28]. According to previous studies, a series of risk factors were associated with the development of IVDD, which included smoking, injury, overweight, and abnormal expression of genes [29, 30, 31, 32]. The causes of IVDD can be categorized as microlevel and macrolevel factors.

**Figure 1:**
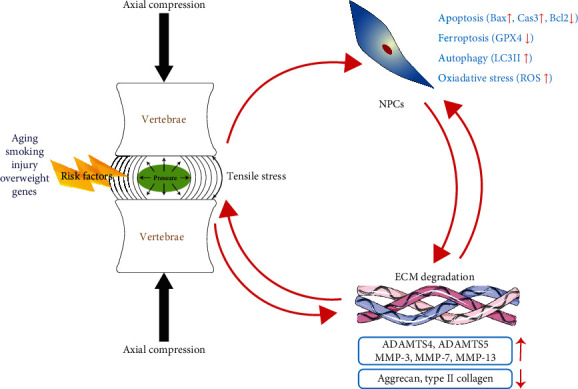
The vicious circle of IVDD. The schematic diagram illustrates the interplay of various factors contributing to IVDD and the roles of NPCs and ECM degradation in this process. The normal function of IVD is dependent on the interactions of cells, ECM, and mechanical forces. If the homeostasis is disrupted, the function of cells in the IVD will be impaired. Aging, smoking, injury, overweight, and genetic predisposition are highlighted as significant risk factors contributing to IVDD. The diagram shows axial compression applied to the vertebrae, creating pressure within the intervertebral disc. Under tensile stress, NPCs are influenced by several degenerative processes, including increased apoptosis (Bax↑, Cas3↑, and Bcl2↓), ferroptosis (GPX4↓), autophagy (LC3II↑), and oxidative stress (ROS↑). The ECM undergoes degradation through the action of various matrix-degrading enzymes, such as ADAMTS4, ADAMTS5, MMP-3, MMP-7, and MMP-13, leading to the breakdown of crucial structural components like aggrecan and type II collagen. Disruption of any of the disc tissues through aging, injury, or chronic mechanical loading will not only alter the mechanical properties but also lead to various pathological events including apoptosis, ferroptosis, autophagy, and oxidative stress of NP and ECM degradation. Then, NPCs will stop produce the main component of ECM such as collagen and aggrecan. This will lead to the reduction in hydrostatic pressure and increase shear forces on the cells. Fluid shifts can be induced by static loads, consequently resulting in the disorder of gene expression, overly activated MMP and cell death. And in turn, these cellular effects can lead to progressive degeneration.

First, from a macro perspective, mechanical load plays an essential regulatory role in the development of IVDD (Figure 1). The close relationship between mechanical loading and IVDD has been well established [33, 34]. At the human body level, spinal force level is dependent on body mass index, external loads, working posture, and muscle function. At the tissue level, stress and strains are distributed in the IVD, which can be converted into biochemical signals and result in cellular responses, such as the compensatory enhancement in cell number, the impaired cell function, and even cell death. At each functional spinal unit, mechanical loads are shared between the anterior IVD and the two posterior facet joints. The main role of IVD is to support compressive forces from diverse activities, and the facets can resist shear during spinal movements [35]. The biomechanical synergy between the disc and facets plays a central role in maintaining the normal function of spinal unit, and the synergy is lost when the disc height and the compliance are affected by various spinal diseases such as IVDD, spinal dysgenesis, and spine fracture. The loss of biomechanical synergy between the disc and facets will, in turn, aggravate IVDD in a manner of vicious circle. What's more, excessive mechanical loading can lead to abnormal shift in cell function. For example, under excessive compression, genes relevant to ECM degradation can be upregulated, which might result in the decrease of IVD height and mechanical instability of spinal unit [36, 37, 38]. In addition, in static compression experiments of IVD, fluid shifts can be induced by static loads, which subsequently results in decreased NP volume [39]. Because fluid is decreased in the NP tissues, the ECM and cells are consolidated [40, 41], which consequently causes the disorder of gene expression, overly activated matrix metalloproteinase (MMPs) and cell death [39, 42, 43]. And in turn, these unfavorable cellular effects will further lead to damaged changes of architecture and mechanics in IVD.

Second, from a microscopic standpoint, the IVDD is characterized by the loss of NP cells and the degradation of ECM, which are caused by complicated pathogenesis including oxidative stress, autophagy, apoptosis, and ferroptosis [6, 8, 44, 45] (Figure 1). An equilibrium between anabolic and catabolic mechanisms is maintained in normal IVD, while shifts toward degradation are primary features of IVDD. The synthesis and breakdown of ECM are promoted by anabolic agents such as catabolic agents such as MMPs and a disintegrin and metalloproteinase with thrombospondin motifs (ADAMTS). Normally, aggrecan and COLII are the major components of ECM in the NP tissue [46]. The levels of aggrecan and collagen within the ECM of IVD is an essential requisite for IVD repair [47]. The hypoxia-inducible factor (HIF)-1*α* can enhance the activity of aggrecan gene promoter and increase the level of aggrecan in NP tissues, allowing the NPCs to function normally under low oxygen tension [23]. Mutation or deficiency of the aggrecan-related genes can cause chondrodysplasias, which affect the development of both hyaline cartilages and IVD. The aggrecan can be cleaved by ADAMTS-4, ADAMTS-5, and MMPs such as MMP-3, MMP-7, and MMP-13 [48]. In the lumbar disc of adults, there are multiple layers of collagen fibers around the central gelatinous NP. Although, the concentration of proteoglycans and water in NP tissues is higher than other part of the IVD, the level of total collagen is the highest in the outer AF but the lowest in the NP [49, 50]. It was reported that the ratio of aggrecan to water in the degenerative NP tissues would decrease, whereas the ratio of collagen to aggrecan would increase, making NP tissue shift from chondrocyte-like to fibrosis-like phenotype [51, 52]. Therefore, the proper level and structure of collagens in the IVD is essential in maintaining the normal function of IVD. However, during the progress of IVDD, one of the most important early changes is the increased level of MMPs and ADAMTS [21]. These abnormal alternations within IVD tissue will in turn contribute to changes of biochemical change, and thereby result in IVDD [21]. In addition, a series of factors could lead to the degeneration of IVD. For example, the dysfunction of NPCs, such as oxidative stress and abnormal autophagy, and excessive cell death including apoptosis and ferroptosis are closely relevant to IVDD. Among the risk factors, the overactivated oxidative stress can result in catastrophic injuries to IVD. It was reported that IVDD could be alleviated by p16 deficiency through suppressing the excessive oxidative stress [9]. And our previous study revealed that the level of ROS was closely associated with IVDD [53]. Besides, Tang et al. [54] reported that the IVDD could be ameliorated by the Keap1/Nrf2/p62 pathway through enhancing the autophagy in NPCs. What is more, both apoptosis and ferroptosis could take place in NPCs and excessive cell death in NPCs could aggravate the degeneration of IVD.

## 3. GSH

**Figure 2:**
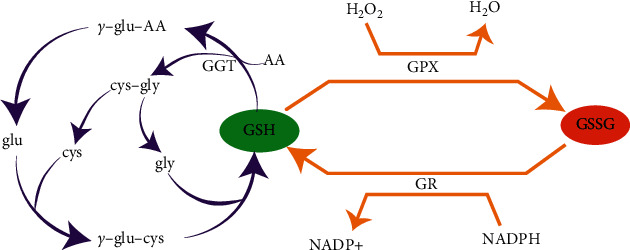
The *γ*-glutamyl cycle and the role of GSH in the redox buffering system. GSH is synthesized from the amino acid precursors glutamate (Glu), cysteine (cys), and glycine (gly) in two steps: first, glutamate combines with cysteine to form *γ*-glutamylcysteine (*γ*-glu–cys) via the enzyme *γ*-glutamylcysteine synthetase; second, *γ*-glutamylcysteine combines with glycine to form GSH via the enzyme glutathione synthetase. The diagram also includes the *γ*-glutamyl cycle, where GSH is broken down into its constituent amino acids by *γ*-glutamyl transferase (GGT), producing *γ*-glutamyl amino acids (*γ*-glu–AA) and cysteinylglycine (cys–gly). These products can be further broken down into individual amino acids, which can reenter the GSH synthesis pathway. GSH can be oxidized to glutathione disulfide (GSSG) in a reaction catalyzed by glutathione peroxidase (GPX) as it reduces hydrogen peroxide (H_2_O_2_) to water (H_2_O). GSSG can then be reduced back to GSH by glutathione reductase (GR) in a reaction that requires NADPH, which is oxidized to NADP+ in the process.

GSH, a cysteine-containing tripeptide, is the most important antioxidant defense and is found at millimolar concentrations in nearly all eukaryotes [55, 56]. Early in 1970, Orlowski and Meister et al. [57] proposed that the *γ*-glutamyl cycle plays a pivotal role in GSH metabolism (Figure 2). Two critical cytosolic enzymes, *γ*-glutamylcysteine synthetase and GSH synthetase, participate in the synthesis of GSH from three amino acids including glutamate, cysteine, and glycine. According to the theory of *γ*-glutamyl cycle, biosynthesis is followed by GSH degradation [57]. The degradation of GSH is catalyzed by *γ*-glutamyl transferase [58]. Being the major source of circulating GSH, liver possesses a large amount of GSH [59]. The kidney, lung, and intestine consume the most liver-derived GSH [60], indicating that the marked oxidative stress might occur in these organs. It is worth noting that in the thin film of liquid on the alveolar surface of the lung, high concentration of GSH can be secreted by epithelial cells [61], which might be associated with the protection against oxidants caused by smoking or inhaled particles.

There are two major forms of GSH: reduced GSH and oxidized glutathione disulfide (GSSG) [62]. In the process of transformation between GSH and GSSG, GSH peroxidases (GPXs) catalyze the oxidation of GSH to form GSSG (Figure 2). ROS can be scavenged by GSH under the catalysis function of GPXs. GPXs catalyzes H_2_O_2_ into H_2_O and GSSG through consuming GSH. And with the help of electrons supplied by NADPH, glutathione reductase can reduce GSSG to GSH [63]. GSH exerts many physiological functions primarily through its redox-active sulfhydryl group (–SH). It was reported that GSH plays an essential role in decreasing oxidative stress, maintaining redox balance, promoting the metabolic detoxification, and regulating immune system [64]. GSH is involved in the removal of peroxides and many xenobiotic compounds by its redox-active sulfhydryl group (–SH), regulating different kinds of programmed cell death including apoptosis, necroptosis, ferroptosis, and autophagy [56]. Because of the essential role of GSH in combating oxidative stress, GSH might be used as a potent therapeutic strategy for IVDD.

## 4. GSH Involved in the Regulation of Various Pathophysiological Processes

### 4.1. GSH Regulates ECM Metabolism

The main structural matrix macromolecules including aggrecan and COLII are essential components of the ECM, which are responsible for the mechanical properties and the microenvironment of the IVD. Catabolism of ECM tends to be enhanced during IVDD, which might cause biomechanical changes. Yang et al. [65] first investigated the effects of GSH on ECM synthesis using human NPCs. The results of the study revealed that GSH significantly inhibited ECM degradation induced by H_2_O_2_ or IL-1*β*, and the levels of COLII and aggrecan were remarkably increased by 49% and 46%, respectively, after the treatment of GSH at the concentration of 1 mM [65]. However, this study was lack of in-depth study on the mechanisms and *in vivo* verification. Besides the protective role in IVDD, in a study of network pharmacology, many target genes were identified for GSH against cleft lip, which can be mainly divided into two gene families including the integrins or MMPs families [66]. These genes play pivotal roles in the developmental processes of the facial structure and the regulation of ECM and fibrillogenesis [66]. Fibrillogenesis is characterized by the development of fine fibrils (collagen) and the binding between fibronectin and integrins, which is regulated by the MMPs. Therefore, it was believed that GSH also has a protective effect against cleft lip. In addition, it was reported that GSH can suppress the degradation of collagen through inhibiting the expression of type Ⅰ plasminogen activator inhibitor [67]. Wei et al. [68] revealed that the activation of Wnt/*β*-catenin signaling pathway could be triggered by the depletion of GSH, causing the increase of type I collagen that is closely associated with fibrosis. Besides, in a study of GSH and citrate-capped copper oxide nanoparticles, the results revealed that the matrix destabilization was inhibited by GSH that promoted the wound healing [69]. In addition, it was reported that the activity of MMP-2 can be activated by peroxynitrite, but this effect can be attenuated by GSH [70]. Consistently, the depletion of GSH accelerated the airspace enlargement of lungs and increased the activities of various MMP isoforms [71]. Taken together, these findings above suggest that GSH can directly or indirectly regulate the synthesis and catabolism of ECM. And GSH may have a therapeutic potential for IVDD by maintaining the balance of ECM synthesis and catabolism. However, the exact mechanisms and the roles of GSH in regulating the subtypes of collagen and MMPs in the development of IVDD remain further investigation.

### 4.2. GSH Inhibits Apoptosis and Ferroptosis

The NP of the disc is crucial to its mechanical function, which is mainly composed of NPCs and ECM [72]. In the process of IVDD, the dysfunction of NPCs can not only induce ECM degradation but also affect cell numbers. Programmed cell death, such as apoptosis and ferroptosis, plays essential roles in reducing the number of functional NPCs and suppressing ECM synthesis [54, 73, 74, 75].

Apoptosis, a programmed cell death process, significantly influences IVDD. Studies have reported an increase in cell apoptosis within IVDD [33], notably observed through elevated levels of the proapoptotic factor Bax and decreased levels of the antiapoptotic factor Bcl-2 in IL-1*β*-treated human NPCs [76]. Furthermore, pro-inflammatory cytokines including IL-1*β* and TNF-*α* could directly induce apoptosis of NPCs [77, 78]. In a previous study, the authors revealed that the exposure of NPCs to pro-inflammatory cytokines promoted DNA damage [79, 80]. The inhibition of excessive apoptosis of NPCs could ameliorate the development of IVDD [81].

Currently, various studies about pharmacological or molecular treatment targeting apoptosis pathway in IVDD have been performed. For example, it was reported that CircERCC2 ameliorated IVDD by repressing apoptosis in human NPCs [82]. Additionally, it was revealed that metformin protected against apoptosis in NPCs and ameliorated IVDD in animal model [22]. It was shown that GSH can significantly protect the NPCs from apoptosis induced by H_2_O_2_ through methods of live/dead and apoptotic staining [65]. But the specific molecular mechanisms of GSH in regulating apoptosis in NPCs are not described in that report. Therefore, further researches about the mechanisms or signaling pathways are required. Interestingly, a study conducted by Kang et al. [83] demonstrated that the cellular apoptosis of NPCs could be ameliorated by lncRNA ANPODRT through activating the Nrf2 signaling pathway that has been reported to be closely associated with the level of GSH [84]. Researchers found that Nrf2 expression was decreased in human NPCs, and that Nrf2 deficiency promoted disc degeneration [85]. Zhang et al. [86] revealed that the H_2_O_2_-induced apoptosis in NPCs could be alleviated by bergenin through promoting the activation of PPAR-*γ* pathway that has also been uncovered to be involved in the regulation of GSH [86, 87]. Therefore, both the Nrf-2 pathway and the PPAR-*γ* pathway could be potential GSH-triggered regulatory mechanisms in protecting NPCs from apoptosis.

**Figure 3:**
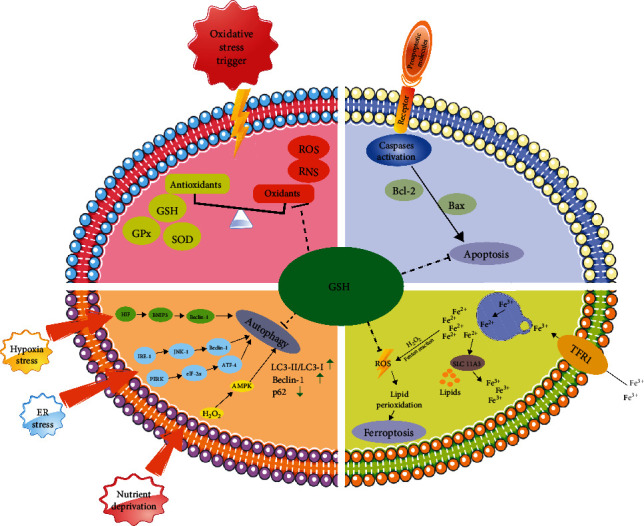
The role of GSH in protecting cells from oxidative stress and its involvement in various cellular processes, including apoptosis, autophagy, and ferroptosis, is presented. Oxidative stress triggers lead to an increase in ROS and RNS, which are counteracted by antioxidants like GSH, glutathione peroxidase (GPx), and superoxide dismutase (SOD). GSH helps regulate apoptosis by influencing the balance between proapoptotic (Bax) and antiapoptotic (Bcl-2) molecules, leading to the activation of caspases and programmed cell death. Under stress conditions, such as hypoxia, endoplasmic reticulum (ER) stress, and nutrient deprivation, GSH is involved in autophagy regulation through pathways, including HIF, BNIP3, Beclin-1, IRE-1, JNK-1, PERK, ATF-4, and AMPK, which induce autophagy markers LC3-II, LC3-I, Beclin-1, and p62. GSH also plays a critical role in preventing ferroptosis, an iron-dependent cell death associated with lipid peroxidation, by mitigating ROS generated via the Fenton reaction.

Researchers have found that the decrease in GSH concentration in cells, which might be ascribed to mechanisms including the direct GSH oxidation promoted by ROS and the GSH export from cells, is an early event in the apoptotic cascade [88, 89, 90]. Besides, depletion of GSH can directly lead to apoptosis in renal collecting duct cells through modulating the activation of TRPM2 cation channel, and the levels of caspase-3 and caspase-9 were also significantly increased [91]. A study conducted by Zhou et al. [92] revealed that GSH protected human brain vascular endothelial cells from apoptosis and oxidative stress via inhibiting the overactivation of Akt pathway [92] that has been reported to be closely associated with the delaying IVDD [93, 94]. Circu and Aw [89] believed that GSH can protect cells from apoptosis by the redox active cysteines at the catalytic sites of caspases. But in response to different stimuli and cell types, the underlying apoptotic pathway and mechanism vary. Taken together, these findings suggested that GSH might be a potential treatment for apoptosis by modulating relevant apoptotic signaling pathways that remain unclear during the process of IVDD (Figure 3).

Ferroptosis is a newly proposed mode of cell death, which mainly involves disorder of iron homeostasis and lipid peroxidation metabolism [95]. The morphology, biochemistry, and genetic characteristics of ferroptosis, a type of programmed oxidative cell death, differs from other cell death forms. Morphologically, ferroptosis is characterized by the decreased mitochondrial volume and mitochondrial cristae and the increased bilayer membrane density, but the cell membrane, the nucleus and chromatin are normal; biochemically, the intracellular GSH and the GPX4 are both decreased, and lipid peroxides increase because of the lack of GPX4; and genetically, ferroptosis can be regulated by various genes [95]. It was reported that specific inhibitors could effectively prevent ferroptosis in the kidney, cerebrum, and other organs [96]. Moreover, studies revealed that ferroptosis correlated closely with IVDD [10, 97]. It was reported that ferroportin had a protective role in NPCs through inhibiting ferroptosis, and ferroptosis may be a target for therapeutic intervention in IVDD [74]. And a study conducted by Zhang et al. [97] demonstrated that the ferroptosis of NPCs was triggered and enhanced by homocysteine through promoting the methylation of GPX4 that has been widely acknowledged to be a key catalytic enzyme in the production of GSH, indicating that GSH could play an important role in protecting NPCs from ferroptosis. What is more, Yang et al. [10] reported that *tert*-butyl hydroperoxide was involved in inducing ferroptosis of AF cells (AFCs) and NPCs through methods of knockdown and overexpression of NCOA4, and the findings revealed that the oxidative stress-induced ferroptosis played a pivotal role in the pathogenesis of IVDD. Li et al. [98] reported that the activation transcription factor 3 promoted the FAoptosis (ferroptosis and apoptosis), and the study revealed that agents suppressing ferroptosis may serve as treatment modalities in IVDD. Consistently, Shan et al. [99] reported that the process of neovascularization in herniated NP can lead to the exposure of NP tissues to high levels of heme and consequently, the degeneration of herniated NP was accelerated. Previous study revealed that the substances inducing ferroptosis can be classified into four categories which mainly reduce GSH levels, inhibit GPX4, degrade GPX4, and inactivate GPX4, respectively [95], indicating that GSH play a central role in suppressing ferroptosis. The inhibitors of ferroptosis exert protective effect by inhibiting accumulation of iron or curbing lipid peroxidation [95]. Chen et al. [100] reported that ferroptosis can be enhanced by the degradation of GSH through the general control nonderepressible 2-eIF2*α*- activating transcription factor 4 pathway. It was believed that the reduced GSH plays a central role in inhibiting ferroptosis through the elimination of oxidized phospholipids that can directly lead to lipid peroxidation via GPX4 [15] (Figure 3). However, studies on the specific mechanisms of GSH in regulating ferroptosis of NPCs are limited. We speculated that GSH might protect NPCs from ferroptosis by eliminating oxidized phospholipids in IVDD. Taken together, the findings above suggested that GSH might play an essential role in curbing the ferroptosis of cells in IVDD, and that ferroptosis could be a target of treatment for IVDD. Nevertheless, studies on the role and mechanism of GSH in regulating the ferroptosis in IVDD are still required.

### 4.3. GSH Inhibits Autophagy

As IVDD proceeds, ECM metabolism will become unbalanced and apoptotic NPCs will increase. The proteins and organelles with abnormalities in structure and function would be degraded by the degradation systems. In cells, there exist two systems of protein degradation: the ubiquitin proteasome system and autophagy [101]. The ubiquitin proteasome system functions through the way of the selective degradation of most proteins. However, the autophagy system can degrade dysfunctional proteins and organelles via both nonselective and selective methods [102]. Autophagy is regarded as a catabolic process in which organelles and cytoplasmic proteins are degraded and recycled in autolysosomes under stress condition. While autophagy tends to be considered as a cell protective mechanism, excessive digestion can trigger autophagic cell death and is regarded as a mechanism of tumor suppressive [103, 104]. The autophagic process is characterized by a series of changes in the membrane structure, which are triggered by activating autophagosomes [105]. The formation of some membrane structures including phagophores, autophagosomes, and autolysosomes are regulated by autophagy-related proteins. Under certain conditions, autophagy that can be classified into selective and nonselective autophagy can regulate cell death or cell survival. IVDD is characterized by abnormal induction of apoptosis and premature senescence of NPCs, the degradation of ECM, and the accumulation of inflammatory cytokines that can promote the degeneration of IVD. Thus, autophagy was regarded as a potential therapeutic modulator in IVDD and other diseases including OA [106, 107]. Various stresses may induce autophagy, such as endoplasmic reticulum (ER) stress and hypoxia stress [108]. ER stress stimulates autophagy through two main pathways, the protein kinase-like ER kinase (PERK) and eukaryotic initiation factor-2*α* pathway, and the inositol requiring enzyme 1/c-Jun N-terminal protein kinase (JNK)1 pathway [108, 109] (Figure 3). Hypoxia stress can activate autophagy through HIF, Bcl2/adenovirus E1B 19 kDa protein-interacting protein 3 (BNIP3), and BNIP3-like protein (BNIP3L) [110]. In addition, nutrient deprivation is also one of the causes of autophagy. It was reported that H_2_O_2_ is produced immediately after starvation [111, 112]. In addition, it was believed that the exposure to H_2_O_2_ may activate AMP-activated kinase that can induce autophagy [113] (Figure 3). What is more, a series of studies revealed that GSH is associated with the inhibition of autophagy in many diseases. Sun et al. [17] reported that the depletion of GSH can trigger autophagy in retinal pigment epithelial cells, which is characterized by the increased level of LC3II. Chen et al. [114] reported that legumain promoted chaperone-mediated autophagy of GPX4 and the deficiency of legumain prevented the autophagy process in acute kidney injury. In addition, GSH can modulate autophagy by transforming the redox conditions to a relatively more oxidizing state if the GSH levels decrease in cancer cells [115, 116]. Ouyang et al. [116] believed that S-adenosylmethionine can block autophagy through enhancing the production of GSH. Various pathways that can reduce intracellular GSH may promote the activation of autophagy. It was reported that the inhibition of the *x*_c_ system via sulfasalazine, which can inhibit the transportation of cystine into tumor cells and thus suppress GSH synthesis, is associated with the activation of autophagy [117]. In summary, all the evidence above suggests that autophagy is associated with GSH and the over-activation of autophagy can accelerate the degeneration of IVD. Although, there is growing evidence for the role of GSH in suppressing autophagy, the mechanism is still elusive and further studies are needed.

## 5. The Relationship between Oxidative Stress and GSH in IVDD

### 5.1. The Role of Oxidative Stress in IVDD

Previous studies have associated elevated ROS in the IVD with disorder of redox homeostasis that can result in the progression of IVD [9]. ROS are erratic and reactive molecules, mainly consisting of superoxide anions (O^2−^), hydroxyl radicals (OH^−^), hydrogen peroxide (H_2_O_2_), and hypochlorite ions (OCl^−^) [118, 119]. In addition, reactive nitrogen species (RNS) including nitric oxide (NO) tends to be considered as members of the ROS family, which are generated in cells by metabolic redox reactions [118]. In IVDD, superoxide anions are produced by the NADPH oxidases family through adding electrons to molecular oxygen [120]. These highly reactive oxidative molecules are potentially damaging to living cells. Oxidative stress is an imbalance between prooxidants and antioxidants [121] (Figure 3). Antioxidants are essential in maintaining human body health. Antioxidant enzymes including superoxide dismutase (SOD), catalase, and GPX are responsible for scavenging H_2_O_2_ and peroxides through interacting with other molecules, such as catalase, thioredoxin, and GSH [44]. And it was reported that exogenous antioxidants including reduced GSH, vitamins C and E can help inhibit the effects of ROS [44, 122]. A previous study reported that the ROS level is significantly correlated with the severity of LBP, and that the ROS levels were significantly increased in puncture-induced IVDD [123]. Feng et al. [124] believed that mitochondrion dysfunction is a core trigger of the production of excessive ROS and that the mitochondrion is an important target of ROS, which might lead to a vicious cycle in IVD. The highly active ROS can interact with intracellular molecules in IVD, and NPCs have antioxidants to neutralize ROS to maintain redox homeostasis. It was reported that the activity of SOD, one of the antioxidants, declined with the development of IVDD in rat lumber discs. Gruber et al. [125] reported that the level of methionine sulfoxide reductase was decreased in human senescent AFCs, which can scavenge ROS through reducing methionine residues. The findings suggest that the decreased antioxidant in degenerated IVD may lead to the accumulation of ROS. It was reported that the increased ROS levels can cause severe oxidative damage to cellular proteins, carbohydrates, and DNA [126], and ultimately result in IVDD pathogenesis. Under the condition of IVDD, the accumulation of ROS may trigger NPCs death and subsequently induce ECM degradation by activating MMPs and ADAMTS.

### 5.2. GSH Protects IVD from Inflammation and Oxidative Stress

**Figure 4:**
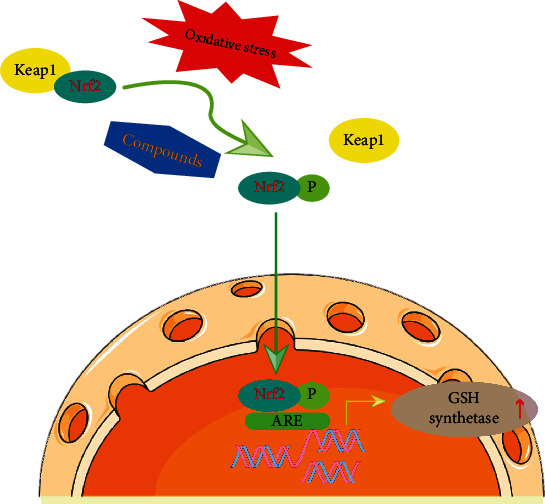
Compounds can raise the level of GSH by activating the Nrf-2 pathway. Under static conditions, Nrf2 was inhibited by Keap1 binding to Nrf-2 in the cytoplasm. But oxidative stress triggers the dissociation of Nrf2 from its inhibitor Keap1. Once released, Nrf2 is phosphorylated (Nrf2-P) and translocates into the nucleus. Inside the nucleus, phosphorylated Nrf2 binds to the antioxidant response element (ARE) in the DNA, inducing the expression of genes responsible for antioxidant defense, including those encoding for GSH synthetase, which leads to increased GSH production. In addition, some compounds can also activate the Nrf2 signaling pathway and lead to the increase in the level of GSH.

The increased level of ROS plays an essential role in age-associated diseases including IVDD, OA, and neurodegenerative diseases. GSH has been shown to exert protective effects on oxidative stress (Figure 3). It was reported that IL-1*β* could induce the production of NO that is one of the RNS and has been reported to be involved in the process of IVDD [127, 128]. In addition, it was reported that pro-inflammatory cytokine IL-1*β* or mechanical load can trigger the degeneration of NPCs or IVDD through ways associated with NO [129, 130, 131]. In a study conducted by Yang et al. [65], data revealed that GSH at the concentration of 1 mM not only did not negatively affect the cell viability of human NPCs but also protect cells from H_2_O_2_-induced cell death such as apoptosis (apoptosis rate: H_2_O_2_ group, 23.82%; H_2_O_2_/GSH group, 6.15%). In addition, the lowered expression levels of aggrecan and COLII caused by H_2_O_2_ in NPCs were significantly increased by GSH treatment (there was a 46% increase in aggrecan and 49% in COLII) [65]. Besides, the increased level of NO induced by IL-1*β* at 10 ng/mL could be significantly decreased by GSH in human NPCs, and the findings indicated that GSH can retard IVDD because of its antioxidative activity [65]. Within certain level of oxidative stress, GSH could directly neutralize oxidative stress products through its sulfhydryl group [132]. But excessive production of oxidative stress such as ROS could lead to extensive GSH consumption and then the level of oxidative stress could not be efficiently controlled. What is more, it was believed that the parenteral nutrition (PN) solution can produce peroxides that may inhibit methionine adenosyl transferase, and PN infusion can cause the decrease of GSH, which might exacerbate oxidative stress [133]. And it was reported that adding GSH to PN can prevent alveolar loss in newborn animals [134]. Consistently, Morin et al. [135] also reported that GSH helped prevent PN-induced oxidative stress and promoted the protein synthesis in the lungs and muscles. Because the development of IVDD is closely associated with the degradation of ECM that is characterized by the decrease of proteins including aggrecan and COLII, the above studies associated with the role of GSH in PN-induced pathological alterations indirectly indicated that GSH could help ameliorate IVDD. And previous study conducted by Li et al. [66] reported that oxidative stress was enhanced in cleft lip and that the GSH treatment can reduce the excessive ROS production in cleft lip. In their study, the common targets of GSH and cleft lip, which included MMP1, MMP3, and MMP9, were obtained using Cytoscape [66], indicating that GSH might be involved in regulating the expression of ECM-related genes. What is more, the increased level of NO is also one of the most important indicators of oxidative stress [136]. Studies revealed that the overexpression of neuronal NO synthase (NOS) or the treatments with NO donors can lead to the increase of intracellular GSH that was essential to prevent neuronal death [137, 138]. Aquilano et al. [139] reported that the decrease of GSH, which can be induced by the treatment with buthionine sulfoximine (BSO) or by fasting, resulted in the raise of NO activity in muscle, and subsequently caused damaging results. ROS and other free radicals including NO generated in the respiratory chain can cause mtDNA damage in IVDD [119]. The enhanced oxidative stress can not only cause mitochondrial disorders but also promote NPCs apoptosis by damaging the cellular integrity [140, 141]. According to previous studies, the Nrf-2 pathway plays a central role in the production of GSH. Steele et al. [84] reported that the Nrf-2 activators increased the level of GSH in a dose-dependent manner. Under static conditions, Nrf2 was inhibited by Kelch-like ECH-associated protein 1 (Keap1) binding to Nrf-2 in the cytoplasm [142]. Stimulated by oxidative stress, cysteine residues within Keap1 are oxidized, resulting in the dissociation of Keap1 from Nrf-2. And Nrf-2 is translocated into the nucleus and then binds to antioxidant response elements (ARE) to promote the expression of antioxidant genes including GSH-related genes (Figure 4) [84]. Importantly, the Nrf-2 signaling pathway has been proved to ameliorate IVDD through regulating cell apoptosis, senescence, ECM metabolism in NP tissues or NPCs [143]. Therefore, we have reason to speculate that GSH might retard the development of IVDD through modulating the Nrf-2 signaling pathway. Collectively, these studies indicated that the GSH intervention targeted against oxidative stress may be a potential treatment for IVDD. But further *in vivo* and *in vitro* experiments are still required.

## 6. Other Potential Roles of GSH in Regulating IVDD

The excessive activation of inflammasome plays an important role in the development of IVDD. It was reported that NLRP3 inflammasome elevated the expression level of IL-1*β* in IVDD, causing the secretion of metalloproteinases and the subsequent degradation of ECM in NPCs [144]. In addition, it was reported that the NLRP3 inflammasome was activated after a sharp decrease in GSH level in macrophage [145]. In their study, the decreased level of GSH caused the dramatical elevation of ROS that resulted in the activation of NLRP3 inflammasome. But at present, comprehensive researches about the roles and underlying mechanisms of inflammasome regulated by GSH is still lacking.

Previous studies reported that ER stress is essential in the development of IVDD and the degeneration of discs can be retarded through suppressing the ER stress [146, 147]. And in a study conducted by Wang et al., the dysfunction of NPCs was dramatically ameliorated by the treatment of dimethyl fumarate that helped decrease the levels of both GSH and ER stress-related markers. GSH's critical role involves its antioxidant capacity to reduce oxidative stress, which is intrinsically linked to ER stress. Oxidative stress can exacerbate ER stress by disrupting protein folding, thereby, amplifying the unfolded protein response and its associated cell death pathways [148]. By mitigating oxidative stress, GSH indirectly suppresses the activation of ER stress [149], helping to maintain cellular integrity and function within IVD. Furthermore, GSH can directly influence the ER stress response by maintaining the redox balance within the ER itself, thus preventing the accumulation of misfolded proteins and reducing the unfolded protein response (UPR) activation [150]. However, the relationship between GSH and ER stress in the IVDD was not explored. This may be a potential research topic in the treatment of IVDD.

## 7. Small Molecule Compounds that Regulate GSH

ROS are involved in a variety of physiological and pathophysiological processes. Balance between the production and scavenging of ROS is essential in maintaining the favorable physiological environment. Oxidative stress is caused by the disorder of oxidation/antioxidant equilibrium state [56]. It was reported that the healthy cells can tolerate mild oxidative stress, but severe oxidative stress beyond the antioxidant capacity can cause damage to molecules, such as lipids, proteins, and DNA, and even can lead to cell death [151]. There are two main approaches to treat oxidative stress: one is to directly inhibit oxidants and the other is to enhance the antioxidant defense system. Playing essential roles in various diseases such as Parkinson's disease, OA and cancer, GSH is considered one of the most important antioxidants against ROS [152, 153, 154]. Horev-Azaria et al. [155] reported that allicin, a small molecule that can permeate the phospholipids bilayers and interact with other compounds to form compounds containing thiol functional groups, raised the GSH level up to eightfold in a concentration and time-dependent manner, and the exposure of endothelial cells to allicin can significantly inhibit oxidative stress. In their study, the increased GSH level helped alleviate the cytotoxicity in vascular endothelial cells. Yet, the specific mechanism involving the protective role of allicin was not elucidated. Han et al. [156] reported that pyrogallol, a kind of superoxide anion generator, dose-dependently reduced the GSH level in calf pulmonary artery endothelial cells and human umbilical vein endothelial cells (HUVEC), which also caused the elevation of the death rate of the involved cells. But the mechanism was not explored or explained. Chen et al. [157] reported that carnosic acid attenuated the 6-hydroxydopamine-induced cell apoptosis through promoting the production of GSH in a time-dependent manner in SH-SY5Y cells. In their study, carnosic acid suppressed the level of proapoptotic factor caspase 3, which was reversed by the treatment of L-BSO (an inhibitor of GSH synthesis). In addition, the p38 MAPK, JNK1/2, and Nrf-2 pathways were involved in the protective effects of carnosic acid. Krämer et al. [158] reported that dimethyl fumarate protected neuron function and decreased brain tissue loss through preventing the traumatic brain injury-induced depletion of GSH in brain. In their study, the GSH level was restored by dimethyl fumarate through the activation of Nrf-2 pathway that has been reported to be closely associated with the antioxidative effect. Steele et al. [84] reported that four known Nrf2 pathway activators, R-*α*-lipoic acid (LA), *tert*-butylhydroquinone (TBHQ), sulforaphane (SFN) and polygonum cuspidatum can raise the level of GSH in astrocytes in a dose-dependent manner. In the study, with the level of GSH increased up to 2.4-fold, SFN exerted the most potent effect. And polygonum cuspidatum increased GSH up to 1.6-fold, TBHQ (1.5-fold), and LA (1.4-fold) [84]. Muangnoi et al. [159] reported that H_2_O_2_-induced ROS production was decreased and the GSH level was elevated by antioxidant Lutein through regulating the phosphorylated MAPKs in human retinal pigment epithelial cells. Li et al. [160] reported that puerarin inhibited the lead acetate-induced oxidative stress in PC12 cells through raising GSH level. In their study, the protective effects of puerarin were blocked by the inhibitors of PI3K/Akt/GSK-3*β* signaling pathway, suggesting that puerarin modulating the expression of GSH through the PI3K/Akt/GSK-3*β* pathway. He et al. [11] reported that the oxidative stress was inhibited induced by H_2_O_2_ in NPCs was ameliorated by melatonin through increasing the GSH level and reducing the ROS. But the involved mechanisms were not explained in their study. And in a study of SD rats, compared with IVDD rats without the treatment of Danshen, Danshen significantly retarded the degeneration of discs through modulating the oxidative stress via increasing the level of GSH [161]. The results of the studies about the small molecule compounds involving in regulating GSH are summarized in the Table 1. Taken together, the GSH system could be a target for the prevention and treatment of IVDD and some compounds can raise the level of GSH through activating the Nrf-2 signaling pathway (Figure 4). Many small molecule compounds can regulate the physiological functions through raising or decreasing the level of GSH. But further studies are still required.

## 8. Current Challenges, Future Perspective, and Conclusion

In light of the extensive review on the multifaceted role of GSH in IVDD, it becomes evident that leveraging GSH's potent antioxidant capabilities offers a promising avenue for therapeutic intervention in the management of IVDD. The body of evidence underscores GSH's critical function in mitigating oxidative stress-induced cellular apoptosis, autophagy, and ferroptosis, thereby, preserving the integrity of the NPCs and ECM. However, translating these findings into clinical practice necessitates further research to overcome challenges, such as the effective delivery of GSH to the avascular IVD, improving its bioavailability, and unraveling the complex mechanisms through which GSH exerts its protective effects. Future research directions should prioritize the development of novel GSH delivery systems, perhaps through advanced nanotechnology or targeted therapy techniques, that ensure GSH reaches the IVD effectively. Additionally, exploring gene therapy approaches to enhance endogenous GSH synthesis within disc cells could provide a sustainable strategy to counteract oxidative damage. Moreover, the potential of GSH to modulate other IVDD-related pathological processes, such as inflammation and ECM degradation, warrants further investigation to understand the full scope of its therapeutic potential. Addressing these research gaps and challenges will not only deepen our understanding of GSH's role in IVDD but also pave the way for the development of innovative GSH-based therapies that could revolutionize the treatment paradigm for IVDD, offering hope to millions suffering from this debilitating condition. In conclusion, the clinical significance and translational potential of GSH in treating IVDD are substantial. By elucidating the protective mechanisms of GSH against oxidative stress-induced cell death and ECM degradation, we can develop novel therapeutic strategies aimed at mitigating IVDD progression. The use of GSH or GSH-boosting compounds could offer a promising approach to enhance disc cell viability, reduce inflammation, and preserve disc integrity, ultimately improving clinical outcomes for patients suffering from IVDD. Future research should focus on translating these findings into clinical applications to provide effective treatments for this debilitating condition.

## Figures and Tables

**Table 1:** Summary of compounds affecting the level of GSH.

Compound	Year	Author	Main effects	Mechanism	Disease/Cell
Allicin [155]	2009	Limor Horev-Azaria	Prevents ROS damage	By upregulating the phase II detoxifying enzymes and increasing the cellular GSH level	Vascular endothelial cells
Adrenomedullin [162]	2009	Su-Mi Kim	Protects against hypoxia/reoxygenation-induced cell death	By suppression of ROS via the GSH and Trx system	A549 cells and HUVEC
Pyrogallol [156]	2010	Yong Hwan Han	Endothelial cell death	GSH depletion	Endothelial cell
Egg yolk peptides [163]	2010	Denise Young	Inhibit intestinal oxidative stress	Increase antioxidants such as GSH and antioxidant enzymes	A porcine model of intestinal oxidative stress
Vanadyl sulfate [164]	2011	Areum Daseul Kim	Increases the level of GSH	Nrf2 activation	Human Chang liver cells
ASSNAC [165]	2011	Nira Izigov	Prevents from oxidative stress	Upregulates cellular GSH	Vascular endothelial cells
Farnesol [166]	2011	Jingsong Zhu	Induces apoptosis	Depletion of intracellular GSH	*Candida albicans*
Carnosic acid [157]	2012	Jing-Hsien Chen	Prevents 6-hydroxydopamine-induced cell death	By increasing GSH level	SH-SY5Y cells
Rice protein [167]	2012	Lin Yang	Attenuating oxidative damage	Regulating GSH metabolism	Rats
Berberine [168]	2012	Thinnakorn Lao-ong	Antioxidative stress	Restore GSH level	Diabetes mellitus
Curcumin [169]	2012	Jianguo Lin	Attenuating oxidative stress	Stimulating synthesis of GSH and suppression of RAGE expression	Hepatic stellate cells
Hesperidin and naringin [170]	2012	Ayman M. Mahmoud	Antidiabetic effects	Attenuate oxidative stress and proinflammatory cytokine production	High fat fed/streptozotocin-induced type 2 diabetic rats
Danshensu-cysteine analog conjugates [171]	2012	Yaoling Jia	Cardiovascular-protective effects	Increase GSH and decrease malondialdehyde	Human umbilical vein endothelial cells
Curcumin [172]	2013	D. JAT	Reduces oxidative damage	By increasing GSH and preventing membrane permeability transition	Brain cells mitochondria
Resveratrol [173]	2013	Chengzhi Chen	Protects against sodium arsenite-induced oxidative damage	Modulation of intracellular GSH homeostasis	Human bronchial epithelial cell
LA/TBHQ/SFN/*Polygonum cuspidatum* [84]	2013	Megan L. Steele	Increases the level of GSH	By activating Nrf2	Human U373 astroglial cells
Acrolein [174]	2014	Mireille M J P E Sthijns	Adaptation to acrolein	By increasing GSH level	Human bronchial epithelial cells
Puerarin [160]	2014	Chengchong Li	Prevention and treatment of chronic diseases related to lead neurotoxicity	Upregulation of GSH levels and nuclear translocation of Nrf2	PC12 cells
Arsenic [175]	2014	Yongyong Hou	Induction of GSH synthesis	By activating mitogen-activated protein kinases	Human hepatocytes
Rooibos tea [176]	2014	In-Sun Hong	Prevent oxidative stress	Regulate GSH metabolism	Chronic psychological stress
Diosbulbin B [177]	2014	Yibo Ma	Induce liver injury	Through oxidative stress	Liver injury
Melatonin [178]	2014	Mohamed A. El-Missiry	Preventing the potential neurotoxicity of bisphenol-A	Ameliorates oxidative stress and apoptosis	Bisphenol-A exposure
Apocynin [179]	2014	Katarzyna Winiarska	Ameliorates the Zucker diabetic fatty state	Improves renal GSH status	Zucker diabetic fatty rats
PS [180]	2014	Seoungwoo Shin	The protection of keratinocytes against UVB-induced injuries	Attenuate cyclobutane pyrimidine dimer formation, GSH depletion, and apoptosis	UVB-induced damage in skin
Yokukansan [181]	2014	Hitomi Kanno	Inhibits glutamate-induced PC12 cell death	Restore the GSH level	PC12 cells
Zinc [182]	2014	Neha Singla	Alleviates aluminum-induced neurodegeneration	Ameliorates oxidative stress	Aluminum-induced oxidative stress and cellular injury in rat brain
D-512 [183]	2015	Chandrashekhar Voshavar	Neuroprotective therapy for Parkinson's disease	Inhibit oxidative stress produced by depletion of GSH	PC12 cells
TBEP [184]	2015	Yuanxiang Jin	Lead to oxidative stress and endocrine disruption	Decrease GSH and increase GSSG	Tm3 leydig cells
Geniposide [185]	2015	Junming Wang	Protects against acute alcohol-induced liver injury	Increase the level of antioxidant enzymes	Liver injury in mice
Crocin [186]	2015	Manel Boussabbeh	Protect against patulin toxicity	By regulating the antioxidants	Patulin-induced neurotoxicity
Biliatresone [187]	2016	Orith Waisbourd-Zinman	Causes mouse extrahepatic cholangiocyte damage and fibrosis	Through decreased GSH and SOX17	Biliary atresia
NCP [188]	2016	Pavla Plačková	Induces cell death	GSH depletion-associated ER stress and mitochondrial dysfunction	CCRF-CEM cells
Alkylating agent [189]	2016	Alfeu Zanotto-Filho	Blocks ER stress-mediated apoptosis	Via control of GSH pools and protein thiol homeostasis	A subset of lung and head and neck carcinomas
Ebselen [190]	2016	Thangamani S	Antifungal activity	Regulating GSH and ROS production	Fungal cells
Dimethyl fumarate [158]	2017	Tobias Krämer	Improves neurological outcome and reduces brain tissue loss	Preservation of brain GSH levels	Traumatic brain injury
Selegiline [191]	2017	Sara A. Wahdan	Protect against 3-NP-induced neurotoxicity	Antioxidant and antiapoptotic actions	Neurotoxicity in rats
Silymarin [192]	2017	Liang Li	Protects against acrylamide-induced neurotoxicity	Increase the level of antioxidants and Nrf2	PC12 cells
Sulforaphane [193]	2018	Jie Liang	Inhibits inflammatory responses	By increasing ROS and depleting GSH	Primary human T-cells
Sulfur [194]	2018	Issa Layali	Triggers oxidative stress	By GSH depletion and altered expression of GSH-related enzymes	Human airways
Tetrachlorobenzoquinone [195]	2018	Zixuan Liu	Resistance to endoplasmic reticulum stress	Regulating GSH synthesis and protein thiol homeostasis	PC12 cells
Estrogen [196]	2018	Jin A. Shin	Neuroprotection against ischemic injury	Increase intracellular GSH	Ischemic reperfusion injury in brain endothelial cells
*N*-butanoyl GSH [197]	2019	Dolores Limongi	Inhibit inflammation	Restore GSH	Inflammation in macrophages
Triptolide [154]	2020	Di Yu	Suppresses IDH1-mutated malignancy	Via Nrf2-driven GSH synthesis	U251 MG, GSC827, GSC923, BT142, TS603, TB096
Xylitol [198]	2020	Nahoko Tomonobu	Induce selective cancer death	Via regulation of the GSH level	Mouse tumor, HEK293T cells, MeWo cells, PANC- 1 cells, OUMS-24 fibroblasts
Vitamin K [199]	2020	Oluwatosin Adebisi Dosumu	Protects against 7,12-dimethylbenz (A) anthracene induced hepatotoxicity	Increase GSH level	Hepatotoxicity in Wistar rats
VP13/47 [200]	2020	Mariarita Spampinato	Impairs NSCLC progression	By decreasing the GSH level	NSCLC cell line (A549)
Lutein diglutaric acid [159]	2021	Chawanphat Muangnoi	Protect against H_2_O_2_-induced oxidative stress	Through the modulation of MAPKs, apoptotic, and antioxidant molecule including GSH	Human retinal pigment epithelial cells
Methanolic leaf extract of *Allium hookeri* [201]	2021	Barsha Deka Prasenjit Ma	Regulate the blood glucose level	Restore the GSH level	Type 2 diabetes
Glycyrrhetinic acid [202]	2021	Yi Wen	Induces oxidative/nitrative stress and ferroptosis	Through activating NADPH oxidases and iNOS and depriving GSH	Triple-negative breast cancer cells
Dexamethasone [203]	2022	Anne von Mässenhausen	Sensitization to ferroptosis	GSH depletion	Acute kidney injury

## Data Availability

The original data for this work are available upon email request to the corresponding author.

## Abbreviations

6-OHDA: 6-hydroxydopamine
ADAMTS: A disintegrin and metalloproteinase with thrombospondin motifs
AF: Annulus fibrosus
AFCs: Annulus fibrosus cells
AMPK: AMP-activated kinase
ASSNAC: S-allylmercapto-N-acetylcysteine
ATF3: Activation transcription factor 3
BNIP3: Bcl2/adenovirus E1B 19 kDa protein-interacting protein 3
BNIP3L: BNIP3-like protein
BSO: Buthionine sulfoximine
CAPEC: Calf pulmonary artery endothelial cells
COLII: Type II collagen
ECM: Extracellular matrix
eIF2*α*: Eukaryotic initiation factor-2*α*
ER: Endoplasmic reticulum
GCL: Glutamate cysteine ligase
GGT: *γ*-glutamyl transferase
GPX: GSH peroxidase
GPX4: Glutathione peroxidase 4
GPXs: GSH peroxidases
GR: Glutathione reductase
GSH: Glutathione
GSSG: Glutathione disulfide
HIF: Hypoxia-inducible factor
HUVEC: Human umbilical vein endothelial cells
IRE1: Inositol requiring enzyme 1
IVD: Intervertebral disc
IVDD: Intervertebral disc degeneration
JNK: c-Jun N-terminal protein kinase
Keap1: Kelch-like ECH-associated protein 1
LA: R-*α*-Lipoic acid
LBP: Lower back pain
MMP: Matrix metalloproteinase
MMPs: Matrix metalloproteinases
NCP: 9-Norbornyl-6-chloropurine
NO: Nitric oxide
NOS: Nitric oxide synthase
NP: Nucleus pulposus
NPCs: Nucleus pulposus cells
OA: Osteoarthritis
PERK: Protein kinase-like ER kinase
PN: Parenteral nutrition
PS: Phloretin 3′,3-disulfonate
RNS: Reactive nitrogen species
ROS: Reactive oxygen species
SAM: S-adenosylmethionine
SFN: Sulforaphane
TBHP: *tert*-Butyl hydroperoxide
TBHQ: *tert*-Butylhydroquinone
TBI: Traumatic brain injury
TGF: Transforming growth factor.
